# Ubiquitin fold modifier 1 activates NF-κB pathway by down-regulating LZAP expression in the macrophage of diabetic mouse model

**DOI:** 10.1042/BSR20191672

**Published:** 2020-01-02

**Authors:** Xiaolei Hu, Hengyan Zhang, Yuan Song, Langen Zhuang, Qingqing Yang, Minglin Pan, Fengling Chen

**Affiliations:** 1 Department of Endocrinology, The First Affiliated Hospital of Bengbu Medical College, Bengbu, Anhui 233004, China; 2 Department of Endocrinology, The Second Affiliated Hospital of Nanjing Medical University, Nanjing, Jiangsu 210011, China; 3 Department of Endocrinology, Shanghai Ninth People’s Hospital, Shanghai Jiao Tong University School of Medicine, Shanghai, China

**Keywords:** Diabetes mellitus, LZAP, NF-κB, Ubiquitin-fold modifer 1

## Abstract

Inflammatory response is closely related with the development of many serious health problems worldwide including diabetes mellitus (DM). Ubiquitin-fold modifer 1 (Ufm1) is a newly discovered ubiquitin-like protein, while its function remains poorly investigated, especially in inflammatory response and DM. In the present study, we analyzed the role of Ufm1 on inflammatory response in DM, and found that the proinflammatory cytokine levels (tumor necrosis factor-α (TNF-α), interleukin-6 (IL-6) and IL-1β) and Ufm1 expression were highly increased both in the peritoneal macrophages of *db/db* mice and Raw264.7 cells induced by lipopolysaccharide (LPS). Western blot and luciferase reporter assay showed that NF-κB pathway was obviously activated in macrophages and the expression of LZAP, an inhibitor of NF-κB pathway, was down-regulated. With the LZAP knockdown plasmid and activation plasmid, we demonstrated that NF-κB/p65 activation was inhibited by LZAP in macrophages. The interaction of Ufm1 and LZAP was further proved with co-immunoprecipitation assay in HEK293 and Raw264.7 cells. The LZAP expression was also related with the presence of Ufm1 demonstrated by Ufm1 knockdown plasmid and activation plasmid. Besides that, we finally proved that the expression and activation of Ufm1 induced by LPS were regulated by JNK/ATF2 and JNK/c-Jun pathway with the use of SP600125. In conclusion, the present study demonstrated that Ufm 1 could activate NF-κB pathway by down-regulating LZAP in macrophage of diabetes, and its expression and activation were regulated by JNK/ATF2 and c-Jun pathway.

## Introduction

Diabetes mellitus (DM) is one of the most prevalent chronic diseases worldwide, and it is a prevalent public health problem [1]. DM is a result of hyperglycemia that is usually caused by the defects of insulin secretion or/and insulin action. Type 2 diabetes (T2D), which is often characterized by insulin resistance and impaired β-cell function, accounts for more than 90% of diabetes patients [2,3]. T2D is a vital risk factor for many other diseases, including stroke [4], cardiovascular diseases and neurological, and psychiatric disorders [5,6]. It is now widely regarded as the main burden on society and severely affects patients’ life.

Recently, researches have reported that low-grade chronic inflammation is closely involved in the development and progression of T2D, and related complication [7,8]. There is an increasing infiltration of macrophages and immune cells in adipose tissue, pancreas, liver, and many other organs in DM [9]. Infiltrated immune cells produce cytokines and chemokines that influence localized and systemic inflammation, and contribute to T2D occurrence by causing insulin resistance [10]. The presence of hyperglycemia will further promote long-term complications of DM in turn.

Endotoxin lipopolysaccharides (LPS) is one of the most common bacterial components that can be absorbed through intestinal [2]. In normal conditions, the body’s immune system responses against the invading microorganisms [11]. However, the intestinal permeability obviously changes in DM patients because of the existence of hyperglycemia, resulting in increased concentration of LPS in the bloodstream [12]. It has been observed that the serum concentrations of LPS and/or LPS-binding protein are higher in diabetic patients in contrast with healthy people [13]. LPS has been shown to initiate immune and inflammatory responses via toll-like receptor 4 (TLR4) [14]. LPS–TLR4 initiated the downstream signaling in macrophages with MyD88-dependent manner, leading to the activation of NF-κB pathway and the transcription of pro-inflammatory mediators, including TNF-α, IL-6, and IL-1β [15]. These inflammation responses may further lead to insulin resistance and DM [16].

Ubiquitin and ubiquitin-like proteins play an important role in many biological processes such as protein degradation, gene expression, signal/transcriptional regulation, cell cycle progression, stress responses, and inflammation [17–19]. Ufm1 is a new member of the ubiquitin-like protein family that has been identified recently, and displays a similar tertiary structure to ubiquitin. Ufm1 is first synthesized as a precursor and cleaved at the C-terminus by two cysteine proteases: UfSP1 and UfSP2. The processed Ufm1 is then activated by E1 enzyme (Uba5) and transferred to the catalytic cysteine of an E2-conjugating enzyme (Ufc1), and covalently attached and modified the target proteins with the help of E3-ligating enzyme (Ufl1) [17]. A recent study had reported Ufm1 as a novel gene that protected against LPS-induced inflammatory responses in endothelial cells by inhibiting the activation of NF-κB signaling pathway [20]. Ufm1 system was also involved in the endoplasmic reticulum (ER) stress. It was demonstrated that Ufm1 was highly expressed in pancreatic β cells resulting in ER stress [21]. Knockdown of Ufm1 increased endoplasmic reticulum stress induced apoptosis in both macrophages and pancreatic cells [21,22]. Despite these researches, the biological function of Ufm1 still remains largely unknown, especially in macrophages and diabetes.

LZAP (also known as C53 protein and CDK5RAP3) is one of the protein targets of Ufm1. LZAP is also a putative tumor suppressor possessing important effects in multiple cell signaling pathways, such as the regulation of NF-κB signaling [23]. It was demonstrated that LZAP inhibited the activation of basal and stimulated NF-kB pathway by directly bounding to RelA and decreasing the phosphorylation of RelA at serine 536. LZAP was also found at the promoter of some NF-kB-responsive genes [23].

As a consequence, we hypothesized that Ufm1 might influence the stabilization of LZAP and increase its degradation by ubiquitination, ultimately give rise to the activation of NF-kB pathway. Therefore, in the present study, we aimed to investigate the role of Ufm 1 and LZAP in the inflammation response in diabetes. Our results showed that Ufm1 expression was significantly increased, while the LZAP expression was markedly decreased in macrophages of DM, and the NF-kB mediated inflammation pathway was obviously activated. In conclusion, the present study demonstrated that Ufm 1 activated NF-κB pathway by down-regulating LZAP in macrophage of diabetes, and its expression and activation were regulated by JNK/ATF2 and c-Jun pathway.

## Methods

### Reagents

Lipopolysaccharide (LPS) and SP600125 were purchased from Sigma-Aldrich (St Louis, MO, U.S.A.). The cell culture Medium (DMEM, MEM, and RPMI 1640), fetal bovine serum (FBS), penicillin–streptomycin (10,000 U/ml) solution, and phosphate-buffered saline (PBS) were purchased from Thermo Fisher Scientific (Waltham, MA, U.S.A.).

Enzyme-linked immunosorbent assay (ELISA) kits for TNF-α (MTA00B), IL-6 (M6000B), and IL-1β (MLB00C) were obtained from R&D Systems (Minneapolis, MN, U.S.A.). The primary antibodies for Ufm1 (ab109305), LZAP (CDK5RAP3) (ab157203), NF-κB p65 (ab16502), p-NF-κB p65 (ab86299), ATF2 (ab47476), p-ATF2 (ab32019), c-Jun (ab32137), p-c-Jun (ab32385), anti-GFP antibody (ab290), and β-actin (ab8226) were from Abcam (Cambridge, U.K.). Anti-Flag antibody (F7425) was form Sigma. Anti-mouse IgG horseradish peroxidase-conjugated secondary antibody (ab97040) was also from Abcam.

### Animals

All the animals’ experiments were done at the Animal Center of The First Affiliated Hospital of Bengbu Medical College, and all animal experimental procedures complied with the guidelines by the Association for Assessment and Accreditation of Laboratory Animal Care International and approved by the Institutional Animal Ethics Committee of The First Affiliated Hospital of Bengbu Medical College (IAUCU number: LAC-2017-0039). Male C57BLKS/J mice (*db/m*) and spontaneously diabetic mice (C57BL/KsJ-*db/db* mice, *db/db*) aged 8 weeks were obtained from Model Animal Research Center of Nanjing University (MARC, Nanjing, China), and housed under standard conditions (12:12 h light–dark cycle, 23 ± 2°C temperature and 60 ± 10% humidity) with free access to water and food.

For ELISA test, blood samples were obtained and centrifuged for 15 min at 3500 *g*. Then, the serum was collected and stored at −80°C for until use.

### Cell preparation and culture

The mouse peritoneal macrophages (MPM) were harvested from the peritoneal cavity of wild mice and *db/db* mice as described previously [22]. Briefly, mice were injected intraperitoneally with 3 ml of 5% thioglycolate (Sigma Aldrich, Louis, MO, U.S.A.) and housed for 3 days. Then, the mice were anesthetized with intraperitoneally with pentobarbital (30 mg/kg) and xylazine (7 mg/kg) followed by cervical dislocation. Animals were confirmed dead when no breathing or heart beat was detected. MPM were collected from the abdomen by lavage with RPMI 1640 medium containing 1% (v/v) penicillin–streptomycin. After washing for one time, MPM were cultured in 10% FBS containing RPMI 1640 medium and incubated at 37°C and 5% CO_2_.

The mouse macrophages cell line (RAW 264.7) and the human embryonic kidney 293 (HEK293) cell line were obtained from the American Type Culture Collection (ATCC, Rockville, MD, U.S.A.). RAW 264.7 cells were cultured in DMEM, and HEK293 cells were cultured in MEM, both supplemented with 10% fetal bovine serum (FBS), and 1% (v/v) penicillin–streptomycin (10,000 U/ml) in 5% CO_2_ incubator at 37°C and 95% humidity.

### Determination of inflammatory cytokine in serum

The inflammatory cytokines levels of TNF-α, IL-6, and IL-1β in serum were measured by ELISA kits according to the manufacturer’s instructions. Enzyme-linked immunosorbent assay (ELISA) kits for TNF-α (MTA00B), IL-6 (M6000B), and IL-1β (MLB00C) were obtained from R&D Systems (Minneapolis, MN, U.S.A.). All experiments were repeated for three times.

### Western blot analysis

Collected cells were collected and lysed for 1 h on ice. The lysates were centrifuged at 13,000 *g* for 15 min, and the supernatant was collected. The contents of protein extracts were determined by BSA assay. Approximately 30 μg/line protein was separated by 10% SDS-PAGE and then electrotransferred to PVDF membranes. Blots were blocked with 5% BSA for 1.5 h at 37°C followed by incubated overnight with the primary antibodies at 4°C. After that, bands were incubated with peroxidase-conjugated secondary antibody for 1 h at room temperature, and then visualized with Tanon 5200 Chemiluminescence imaging system. The amounts of the target proteins were analyzed using ImageJ analysis software and normalized according to control. Results were obtained from three independent experiments. And all information of primary and secondary antibodies used were described above in regents section.

### Real-time quantitative PCR

Total RNA was extracted from collected MPM and RAW264.7 cells using TRIzol reagent (Invitrogen, Carlsbad, CA, U.S.A.) according to the manufacturer’s protocol. cDNA was then synthesized using the GoScript™ Reverse Transcription kit (Madison, WI, U.S.A.). Quantitative real-time PCR (q-PCR) was carried out according to the protocol of GoTaq® qPCR Master mix kit (Madison, WI, U.S.A.). The relative amount of each gene was determined using 2^−ΔΔ*C*^_T_ method by normalizing target gene *C*_t_ values to those for GAPDH (Δ*C*_t_).

### Knockdown and overexpression assay

RAW264.7 cells were transfected with CDK5RAP3 shRNA Plasmid (m) (sc-108060), UFM1 shRNA Plasmid (m) (sc-154891-SH), and Control shRNA Plasmids (sc-108060) (Santa Cruz Biotechnology) with transfection reagent (Santa Cruz Biotechnology) to decrease the expression of LZAP and Ufm1 separately according to the shRNA transfection protocol of Santa. CDK5RAP3 CRISPR activation plasmid (m) (sc-429782-ACT), UFM1 CRISPR activation plasmid (m) (sc-426807-ACT), and Control CRISPR activation plasmid (sc-437275) (Santa Cruz Biotechnology) were also transfected to cells to overexpress LZAP and Ufm1 separately following the manufacturer’s instructions.

### NF-κB reporter assay

The NF-κB Reporter kit (60614, BPS Bioscience, U.S.A.) is used to monitor the activity of the NF-κB signaling pathway in the cultured cells according to the manufacturer’s protocol. Briefly, NF-κB luciferase reporter vector, firefly luciferase vector (non-inducible luciferase vector), and Renilla luciferase vector were correspondingly transfected into RAW264.7 cells with Lipofectamine™ 2000 (11668027, Invitrogen, Carlsbad, CA, U.S.A.). After 24 h of transfection, cells were treated with LPS for additional 12 h. Then, cells were lysed, and the luciferase activity was measured by Dual Luciferase assay system (60683, BPS Bioscience, U.S.A.) following the protocol.

### Construction of UFM1 promoter reporters and transfections

The construction of UFM1 promoter reporters was performed as described previously [24]. Briefly, the wild-type and mutant UFM1promoter (c.-1889 to c.-1 of NM_001286704.1) were cloned into the pNL1.1 reporter (Promega, Madison, WI, U.S.A.) using the infusion protocol (Clontech, Mountain View, CA). After then, the UFM1 promoter reports, and pNL1.1 empty vector was transfected in RAW264.7 cells. The pGL3 plasmids (Promega, Madison, WI, U.S.A.) that express firefly luciferase driven by the SV40 promoter (Promega, Madison, WI, U.S.A.) were also transfected to normalize the transfection differences. LPS and SP600125 were added to cells 24 h after the transfection and cultured for another 12 h. Then, cells were lysed, and the firefly luciferase and nano-luciferase activity were measured by microplate reader (Perkin-Elmer Life Sciences, Waltham, MA, U.S.A.) following the manufacturer’s protocol.

### Co-immunoprecipitation assays

Co-immunoprecipitation (Co-IP) assay was performed to investigate the *in vivo* binding of LZAP and Ufm1. The expression plasmids encoding Flag tagged Ufm1 and GFP tagged LZAP were transiently transfected into HEK293 and RAW264.7 cells. Cells were harvested and lysed with 0.5 ml of lysis buffer (P0013, Beyotime, Shanghai, China) for 15 min on ice. Cell lysates were centrifugated at 13,000 rpm for 15 min, the supernatant was incubated with the anti-Flag antibody at 4°C for 2 h, and then precleared with protein-A/D-Sepharose (Bioworld Technology Inc., California, U.S.A.) at 4°C overnight. Immunoprecipitated complex was washed three times with lysis buffer and then boiled in SDS sample buffer for 5 min. After then, the immunoprecipitated complex was used in Western blot assay with anti-GFP antibody, followed by peroxidase-conjugated appropriate secondary antibody and visualized by the ECL detection system (Bio-Rad, U.S.A.).

### Statistical analysis

All experiments in the present study were performed at least in triplicate independently. Values were represented as means ± SDs. Comparisons were made using one-way analysis of variance (ANOVA) and Student’s *t* test by GraphPad Prism Software (GraphPad Inc., La Jolla, CA, U.S.A.). *P < 0.05, **P < 0.01 were regarded as statistically significant.

## Results

### Expression of inflammation cytokines and Ufm1 were increased in *db/db* mice and LPS-induced Raw264.7 cells

**Figure 1:**
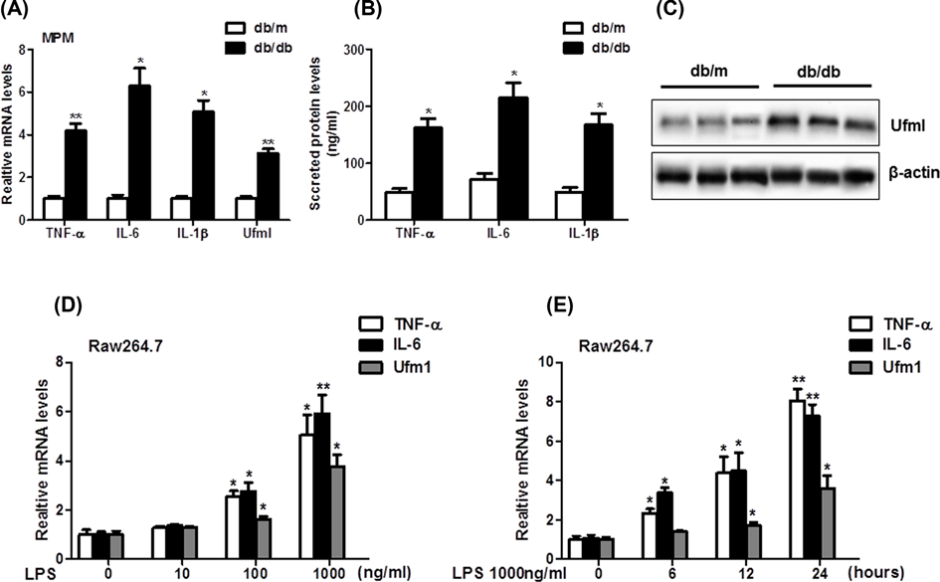
Expression of inflammation cytokines and Ufm1 in *db/db* mice and LPS-induced Raw264.7 cells (**A**) The mRNA levels of TNF-α, IL-6, IL-1β, and Ufm1 in MPMs of *db/m* and *db/db* mice were tested by real-time PCR. (**B**) The secreted proteins levels of TNF-α, IL-6, and IL-1β in serum of *db/m* and *db/db* mice were tested by ELISA. (**C**) The protein expression of Ufm1 in MPMs of *db/m* and *db/db* mice were tested by Western blot. (**D**) Raw264.7 cells were treated with LPS (10, 100, and 1000 ng/ml), and the mRNA levels of TNF-α, IL-6, and Ufm1 were tested by real-time PCR. (**E**) Raw264.7 cells were treated with LPS (6, 12, and 24 h), and the mRNA levels of TNF-α, IL-6, and Ufm1 were tested by real-time PCR. Similar results were obtained in three independent experiments, and one of three representative experiments was shown. The values presented were the mean ± SEM; *P < 0.05, **P < 0.01 versus the control group.

To investigate the role of Ufm1 in diabetes mice, we first analyzed the expression of Ufm1 and proinflammation cytokines (TNF-α, IL-6, and IL-1β) in *db/db* mice. The TNF-α, IL-6, and IL-1β mRNA levels in MPMs (Figure 1A), and protein expression levels in serum (Figure 1B) were significantly increased compared with *db/m* mice. Both mRNA and protein levels of Ufm1 in the MPMs (Figure 1A,C) from the db/db mice were also higher than those from the db/m mice. These results demonstarte the Ufm1 expression and inflammation levels increased in diabetic model mice. In LPS-induced Raw264.7 cells, the TNF-α, IL-6, and Ufm1 mRNA levels were obviously increased in dose-dependent and time-dependent way (Figure 1C,D). The results showed that the expression of inflammation cytokines and Ufm1 in *db/db* mice and LPS-induced Raw264.7 cells were both up-regulated.

### The activation of NF-κB pathway was inhibited by LZAP

**Figure 2:**
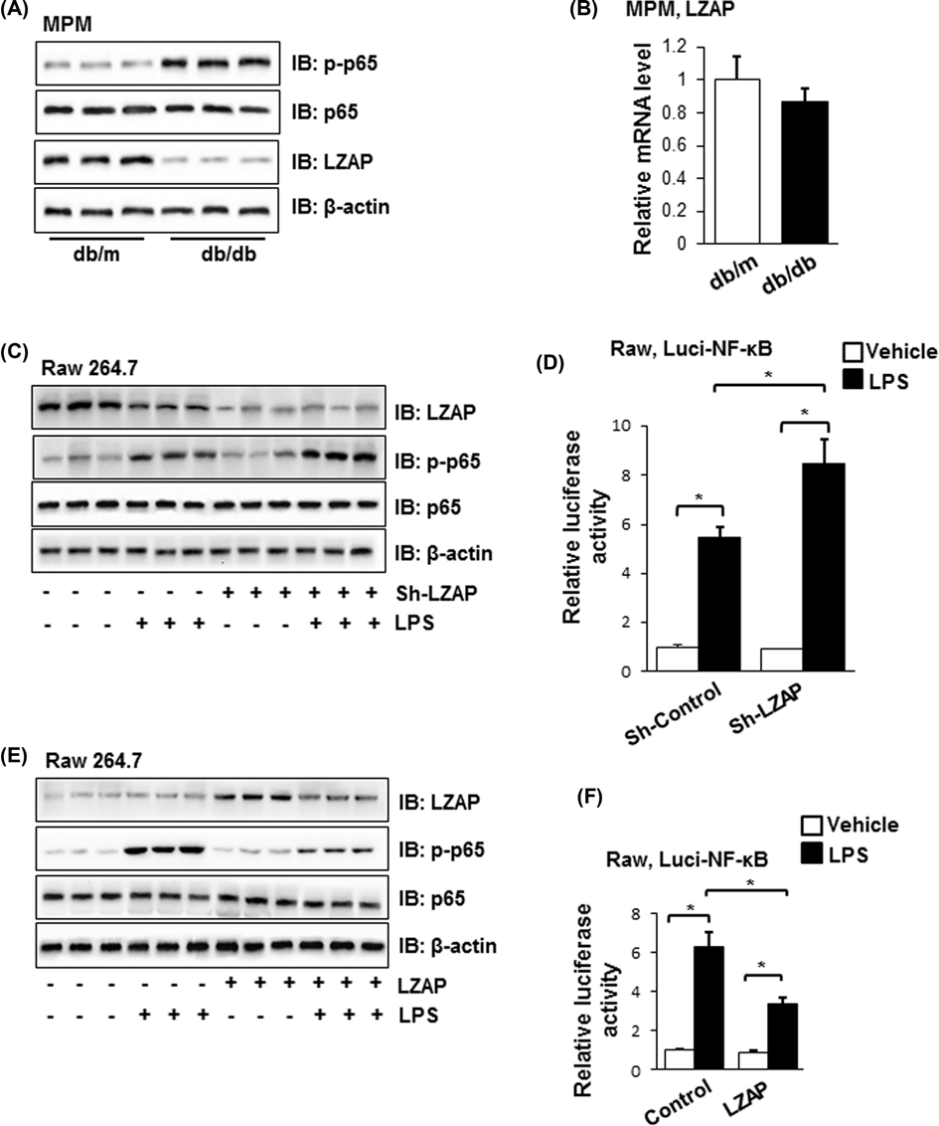
The relation between the expression of LZAP and the activation of NF-κB pathway (**A**) The proteins levels of p-p65, p65, and LZAP in MPMs of *db/m* and *db/db* mice were tested by Western blot. β-Actin acted as a loading control. (**B**) The mRNA levels of LZAP in MPMs of *db/m* and *db/db* mice were tested by real-time PCR. (**C**) Raw264.7 cells were treated with sh-LZAP plasmid and LPS, and the proteins levels of p-p65, p65 and LZAP were tested by Western blot. β-Actin acted as a loading control. (**D**) The activation of NF-κB was tested by luciferase reporter assay with the treatment of sh-LZAP plasmid and LPS. (**E**) Raw264.7 cells were treated with LZAP overexpressed plasmid and LPS, and the proteins levels of p-p65, p65 and LZAP were tested by Western blot. β-Actin acted as a loading control. (**F**) The activation of NF-κB was tested by luciferase reporter assay with the treatment of LZAP overexpressed plasmid and LPS. Similar results were obtained in three independent experiments, and one of three representative experiments was shown. The values presented were the mean ± SEM. *P < 0.05 versus the control group.

The Western blot results in Figure 2A showed that the p-p65 was highly expressed in MPMs of *db/db* mice compared with that in *db/m* mice, while the LZAP protein level was down-regulated. There was no significant difference of the LZAP mRNA levels in MPMs between *db/db* mice group and control group (Figure 2B). To investigate the relation of LZAP and NF-κB pathway, LZAP shRNA and activation plasmids were used in LPS-induced Raw264.7 cells. As we could see in Figure 2C,D, the LZAP was down-regulated in LPS-induced Raw264.7 cells transfected with LZAP shRNA plasmid, and as a result, the protein expression of p-p65 was up-regulated and the relative luci-NF-κB activity increased. In contrast, when LPS-induced Raw264.7 cells transfected with LZAP plasmid, the p-p65 protein expression and the luciferase activity of NF-κB were inhibited (Figure 2E,F). The results demonstrated that the activation of NF-κB pathway was inhibited by LZAP in LPS-induced Raw264.7 cells.

### Ufm1 interacted with LZAP and formed a large complex

**Figure 3:**
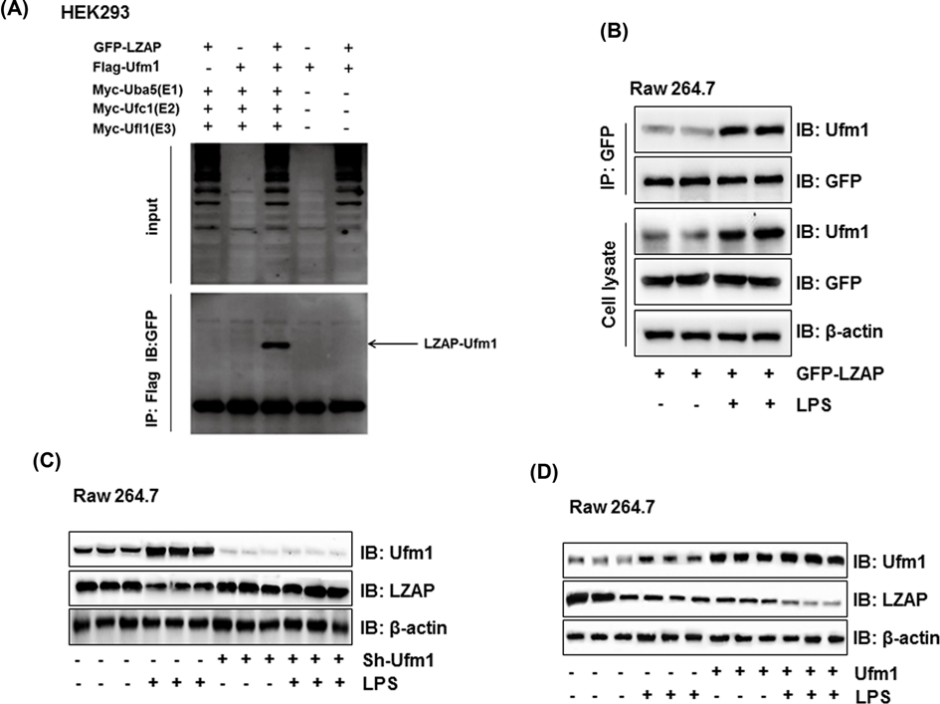
The interaction of Ufm1 and LZAP (**A**) GFP-LZAP, Flag-Ufm1, Myc-Uba5 (E1), Myc-Ufc1 (E2), and Myc-Ufl1 (E3) vectors were transfected in HEK293 cells. The cell lysates were immunoprecipitated (IP) with anti-Flag antibody, followed by immunoblot analysis with anti-GFP antibodies. The arrowheads indicated GFP-LZAP and Flag-Ufm1conjugate. (**B**) GFP-LZAP and Flag-Ufm1 vectors were transfected in Raw264.7 cells. The cell lysates were IP with anti-GFP antibody, followed by immunoblot analysis with anti-Flag or anti-GFP antibodies. (**C**) Raw264.7 cells were treated with sh-Ufm1 plasmid and LPS, and the proteins levels of Ufm1 and LZAP were tested by Western blot. β-Actin acted as a loading control. (**D**) Raw264.7 cells were treated with Ufm1 overexpressed plasmid and LPS, and the proteins levels of Ufm1 and LZAP were tested by Western blot. β-Actin acted as a loading control. Similar results were obtained in three independent experiments, and one of three representative experiments was shown.

To study the interaction between Ufm1 and LZAP, the co-IP assays using overexpressed proteins in HEK293 cells were performed. As shown in Figure 3A, when Myc-Uba5 (E1), Myc-Ufc1 (E2), and Myc-Ufl1 (E3) were present, GFP–LZAP was co-immunoprecipitated with Flag-tagged Ufm1 immunoprecipitate. When Ufm1 and LZAP were overexpressed in Raw264.7 cells, Ufm1 was also co-immunoprecipitated with GFP-LZAP with or without the treatment of LPS (Figure 3A). Besides, LPS treatment significantly increased the co-immunoprecipitated Ufm1 protein level (Figure 3B). Ufm1 shRNA plasmid and activation plasmid were further used in LPS-induced Raw264.7 cells to investigate the regulation of Ufm1 on LZAP. As shown in Figure 3C, Ufm1 protein expression was increased and LZAP protein expression was decreased with the treatment of LPS. While when Ufm1 was knocked down with Ufm1 shRNA plasmid, LPS could not down-regulate the protein expression of LZAP. In contrast, when Ufm1 was overexpressed, the LZAP protein levels were further decreased with or without LPS treatment (Figure 3D). In conclusion, the results proved that Ufm1 interacted with LZAP and decreased the protein expression of LZAP.

### Ufm1 was regulated by JNK/ATF2 and c-Jun

**Figure 4:**
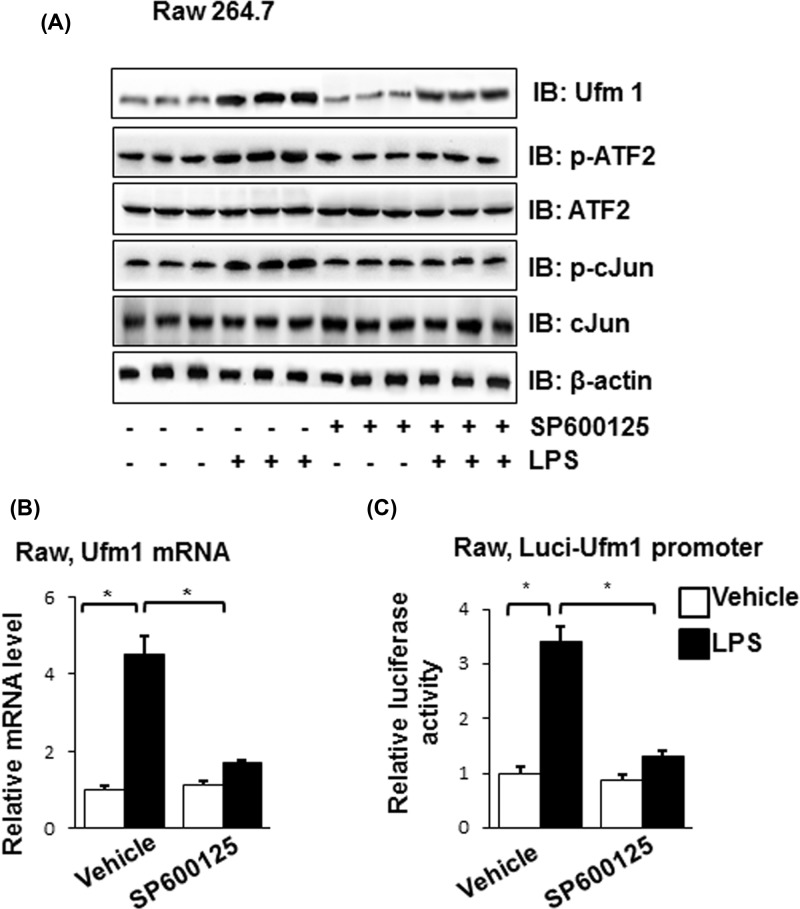
The regulation of Ufm1 by JNK/ATF2 and c-Jun (**A**) SP600125 and LPS were treated to Raw264.7 cells correspondingly the proteins levels of Ufm1, p-ATF2, ATF2, p-c-Jun and c-Jun were tested by Western blot. β-Actin acted as a loading control. (**B**) SP600125 and LPS were treated to Raw264.7 cells, and the Ufm1 mRNA level was detected by real-time PCR. (**C**) SP600125 and LPS were treated to Raw264.7 cells, and the Ufm1 promoter activity was tested by luciferase reporter assay. Similar results were obtained in three independent experiments, and one of three representative experiments was shown. The values presented were the mean ± SEM. *P < 0.05 versus the control group.

As we all know, LPS could activate JNK pathway and the ATF2 and c-Jun are regulated by JNK. We further demonstrated if the increase of Ufm1 induced by LPS was regulated by JNK/ATF2 and c-Jun with the use of SP600125, a known JNK inhibitor. As was shown in Figure 4A, the protein levels of p-ATF2, p-c-Jun and Ufm1 in Raw264.7 cells induced by LPS were decreased by SP600125. Through the real-time PCR and luciferase assay, we found the Ufm1 mRNA level and the Ufm1 promoter activity were also down-regulated by SP600125 (Figure 4B,C). The results showed that expression of Ufm1 was regulated by JNK/ATF2 and c-Jun.

## Discussion

In the present study, we investigated the expressions of Ufm1 and inflammation response in MPMs of diabetic mice and LPS-induced macrophages. We found that the mRNA and protein levels of Ufm1 were significantly increased in MPMs of *db/db* mice and LPS-induced Raw264.7 cells. At the same time, the inflammatory cytokines, including TNF-α, IL-6 and IL-1β, were also up-regulated. Thus, we further discussed if the inflammation response was regulated by Ufm1 in DM.

DM and T2DM in particular is an increasingly common problem worldwide with highly morbidity and mortality [25]. Epidemiologic studies have demonstrated the relation of inflammatory biomarkers and the pathological process of T2DM and complications [8]. Chronic inflammation in DM increases the occurrence of T2DM through insulin resistance and in turn leads to the presence of hyperglycemia to promote long-term complications of diabetes [9]. Although the inflammatory process in diabetes is systemic, the immune cells, especially macrophages in both the adaptive and innate immunity, seem to be more involved [10,26]. Inflammatory markers, such as the pro-inflammatory cytokines TNF-α and IL-6, are the major contributor to local and systemic insulin resistance [27]. These inflammatory cytokines are implicated in the suppression of adiponectin expression in human adipocytes. Low expression of adiponectin are associated with an increased risk for incident T2DM [28]. IL-1β has also been proved to play a central role in energy metabolism and glucose homeostasis. The overexpression of IL-1 members is also connected with the occurrence of diabetes [29]. Our results further demonstrated the increased expression of the inflammatory cytokines in *db/db* mice.

One of the main triggers of inflammation in diabetes is the gut microbiota, such as LPS [30]. LPS is the inflammatory component of the cell wall of the gram-negative bacteria. Researches have shown that LPS level in diabetic patients is higher than health people [11]. LPS interacts with the immune system and then induces a series of inflammatory response [9,31]. Therefore, LPS-induced Raw264.7 cells were used as a cell model in our study to imitate the inflammation in diabetes. As expected, the expression of TNF-α, IL-6, and IL-1β were increased by LPS in Raw264.7 cells. Therefore, for convenience, we studied specific molecular mechanisms in LPS-induced RAW264.7 cells. However, this research method has its limitations, and in future research, we will further verify this mechanism in RAW264.7 cells only under high glucose condition.

NF-κB is known as a major transcription factor that regulates genes responsible for the innate as well as adaptive immune response. The activation of NF-κB pathway plays an important role in the inflammatory process [32]. LZAP has been reported as a putative tumor suppressor dependent on the regulation of NF-κB pathway. Decreased LZAP promoted cellular transformation, enhanced the xenograft tumor growth and angiogenesis [23]. However, the regulation of LZAP on NF-κB in MPMs of diabetes hasn’t been fully studied. Our results showed that the protein expression of LZAP was significantly decreased in MPMs of *db/db* mice and LPS-induced Raw264.7 cells. Meanwhile, the p-p65 protein level was markedly increased indicating that NF-κB pathway was activated in these macrophages. Interestingly, when LZAP expression was further knocked down by sh-RNA, the NF-κB pathway was activated to a higher degree in LPS-induced Raw264.7 cells. In contrast, once the LZAP was up-regulated, the activation of NF-κB pathway was also inhibited. While no significantly LZAP mRNA level change was observed in MPMs of *db/db* mice compared with *db/m* mice. Thus, it was speculated that LZAP was regulated at the protein level. Our results demonstrated the inhibition of LZAP on NF-κB activation in macrophages.

Ufm1 is a newly identified ubiquitin-like protein modifiers with 85 amino acid residues [33]. It has been reported that Ufm1 is up-regulated by ER stress induced diseases such as Type 2 diabetes and ischemic heart injury [34,35]. Zhang et al*.* showed that C53/LZAP protein and the Ufm1-specific E3 ligase RCAD/Ufl1 were involved in ufmylation of endogenous Ufm1 targets [36]. While the ufmylation by Ufm1 for LZAP still need more study. To further investigate the regulation of Ufm1 on LZAP, we performed the co-IP assays in both HEK293 and LPS-induced Raw264.7 cells. The results showed that Ufm1 and LZAP interacted with each other. Down-regulation of Ufm1 increased the accumulation of LZAP in LPS-induced Raw264.7 cells, while Ufm1 overexpression resulted in the obviously reduction of LZAP protein. These results suggested that Ufm1 and LZAP may be contacted and form protein complex. The up-regulated Ufm1 levels in diabetes may decrease the expression of LZAP leading to the activation of NF-κB pathway.

ATF2 and c-Jun, both as an important member of activating protein-1(AP-1), often functions a c-Jun-ATF2 heterodimers and are involved in many cellular processes [37–39]. ATF2 and c-Jun could be phosphorylated by three major MAPKs, including JNK, extracellular signal-regulated kinase (ERK) and p38 in mammals [40]. Our study also tested if the increased Ufm1 expression induced by LPS was regulated by JNK/ATF2 and c-Jun with SP600125, the inhibitor of JNK. As the results shown, the p-ATF2 and p-c-Jun proteins expression were significantly inhibited by SP600125; meanwhile, both the protein and mRNA levels of Ufm1, as well as the Ufm1 promoter activity were obviously down-regulated in LPS-induced Raw264.7 cells.

In conclusion, the present study demonstrated that LPS induced the overexpression of Ufm1 through JNK/ATF2 and c-Jun pathway; the highly expressed Ufm1 further activated the NF-κB pathway and resulted in inflammatory response by decreasing LZAP expression in macrophage of diabetes. Our results provided a new insight to treat with the inflammation in diabetes.

## Abbreviations

Co-IP: co-immunoprecipitation
DM: diabetes mellitus
IL-6: interleukin-6
LPS: lipopolysaccharide
T2D: Type 2 diabetes
TLR4: toll-like receptor 4
TNF-α: tumor necrosis factor-α
Ufm1: Ubiquitin-fold modifer 1
